# Fluoroscopy-Guided High-Intensity Focused Ultrasound Neurotomy of the Lumbar Zygapophyseal Joints: A Clinical Pilot Study

**DOI:** 10.1093/pm/pnab275

**Published:** 2021-09-17

**Authors:** Jordi Perez, Michael Gofeld, Suzanne Leblang, Arik Hananel, Ron Aginsky, Johnny Chen, Jean-Francois Aubry, Yoram Shir

**Affiliations:** 1 McGill University, Montreal, Quebec, Canada; 2 Silver Centre for Pain Care, Toronto, Ontario, Canada; 3 Focused Ultrasound Foundation, Charlottesville, Virginia, USA; 4 FUSMobile, Inc., Alpharetta, Georgia, USA; 5 Georgia Institute of Technology, Atlanta, Georgia, USA; 6 Physics for Medicine Paris, Inserm, ESPCI Paris, CNRS, PSL Research University, Paris, France

**Keywords:** Zygapophyseal Pain, Ablation, Neurotomy, High-Intensity Focused Ultrasound

## Abstract

**Objective:**

To investigate the safety and feasibility of a fluoroscopy-guided, high-intensity focused ultrasound system for zygapophyseal joint denervation as a treatment for chronic low back pain.

**Methods:**

The clinical pilot study was performed on 10 participants diagnosed with lumbar zygapophyseal joint syndrome. Each participant had a documented positive response to a diagnostic block or a previous, clinically beneficial radiofrequency ablation. For a descriptive study, the primary outcome was the safety question. All device- or procedure-related adverse events were collected. Secondary outcome variables included the average numeric rating scale for pain, the Roland-Morris Disability Questionnaire, the Brief Pain Inventory, the Patient Global Impression of Change, the morphine equivalent dose, and the finding of the neurological examination.

**Results:**

All participants tolerated the procedure well with no significant device- or procedure-related adverse events; there was one episode of transient pain during the procedure. The average numeric rating scale score for pain decreased from 6.2 at baseline to 2.1 (n = 10) after 1 month, 4.9 (n = 9) after 3 months, 3.0 (n = 8) after 6 months, and 3.0 (n = 6) after 12 months. The ratio of participants who were considered a treatment success was 90% at 1 month, 50% at 3 months, 60% at 6 months, and 40% at 12 months.

**Conclusions:**

The first clinical pilot study using a noninvasive, fluoroscopy-guided, high-intensity focused ultrasound lumbar zygapophyseal neurotomy resulted in no significant device- or procedure-related adverse events and achieved clinical success comparable with that of routine radiofrequency ablation.

## Introduction

Nearly 80% of adults suffer from low back pain during their lifetimes, and both clinical and research attention has been focused on establishing effective treatment and management strategies [1]. Although the etiology of back pain is often multifactorial, 5% to 45% of cases are related to chronic zygapophyseal joint inflammation and degenerative changes [2–4]. Zygapophyseal joint syndrome is customarily diagnosed by means of analgesic blockade of the afferent loop at the level of the lumbar medial branches (MBs) of the primary dorsal rami or at the level of L5, the primary dorsal ramus itself. After a positive anesthetic test, radiofrequency ablation (RFA) is performed to provide long-lasting pain relief [5, 6]. Although RFA is minimally invasive, it is not devoid of side effects and complications, including aggravation of pain, infection, bleeding, and thermal injury to the exiting nerve root. In addition, special precautions are recommended for patients receiving anticoagulation therapy and those with implanted electrical devices or metallic hardware.

A quest for a different modality is ongoing. One of the potential methods is high-intensity focused ultrasound (HIFU). HIFU is a noninvasive thermal ablation method that concentrates multiple ultrasound beams onto a predetermined target, much like a magnifying glass that converges light on a single point to ignite a fire [7]. The delivery of acoustic energy via sonication generates bioeffects, such as coagulative necrosis, only at the focus, while sparing near- and far-field tissue. Commercially available HIFU devices use either ultrasound or magnetic resonance imaging guidance [8]. HIFU has received regulatory approval for the thermal ablation of soft tissues, including symptomatic uterine fibroids and liver, pancreas, breast, and prostate malignancies, as well as for brain thalamotomy [9]. Beyond thermal ablation of soft tissues, HIFU is also approved to treat pain from bone cancer, bone tumors, multiple myeloma, neuropathic pain, facet arthritis, osteoid osteoma, pancreatic tumors, and soft tissue injuries [9]. A magnetic resonance imaging–guided focused ultrasound (MRgFUS) device has obtained the Conformité Européenne (CE) mark for treating zygapophyseal back pain; however, clinical adoption has been challenging, likely because of high cost, reimbursement hurdles, and a cumbersome and lengthy procedural routine inside the magnetic resonance imaging scanner. Conceptually, clinical MRgFUS procedures target the distal MB and the joint itself, which contradicts the recommended proximal part of the MB for RFA [10]. In a preclinical model, Kaye et al. reported safety and efficacy when MRgFUS was used to target the proximal MB, which resides in a predictable location within 200 micrometers of the bone at the junction of the transverse process and the superior articular process [11]. A fluoroscopy-guided HIFU device that used guidance similar to RFA would have potential advantages over RFA by eliminating invasiveness and would have an advantage over MRgFUS by lowering the procedural cost and reducing procedural time. Therefore, the present study was conducted to evaluate and report the outcomes of the first-in-human fluoroscopy-guided HIFU neurotomy of proximal MBs.

## Methods

**Figure 1.**
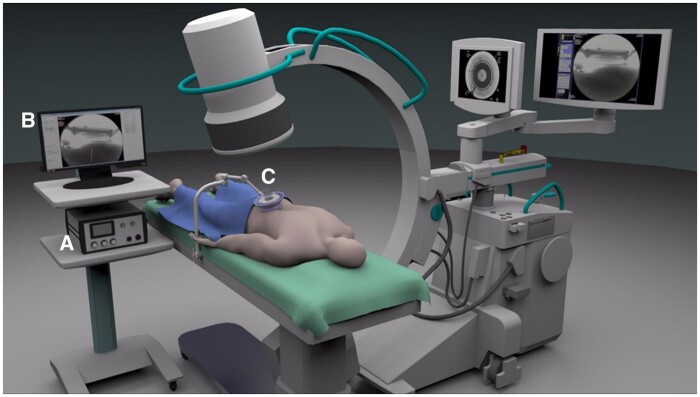
The FUSMobile, Inc. Neurolyser XR, a Fluoroscopy-guided, 1-MHz HIFU Device. **(A)** Power unit, **(B)** fluoroscopic image display, and **(C)** transducer placed over target location .

The study was approved by Health Canada (Investigational Testing Authorization number 264592) and the McGill University and Veritas, Inc. ethical review boards and was registered at ClinicalTrials.gov (NCT03321344). Ten participants with zygapophyseal joint syndrome were recruited between December 2017 and February 2019.The procedures were performed at the McGill Center for Innovative Medicine (JP) in Montreal and at Silver Pain Centre (MG) in Toronto. The investigational device is shown in Figure 1 (Neurolyser XR, FUSMobile, Inc., Alpharetta, GA, USA; fluoroscopy-guided 1 MHz HIFU device).

### Inclusion/Exclusion Criteria

Patients with chronic lumbar zygapophyseal joint syndrome pain lasting more than 6 months qualified for participation in this study. Eligibility was based on a documented positive (greater than 70% pain relief) response to a single anesthetic (diagnostic) block within the previous 12 months or a positive (greater than 70% pain relief) response lasting more than 3 months after RFA performed within the previous 12 months. All participants must have had an average pain rating score of 4 or higher on a 10-point numeric rating scale (NRS) in the month before the study procedure. Inclusion and exclusion criteria are listed in Table 1. The exclusion criteria closely followed the equivalent RFA studies. For example, patients with previous spinal surgery, implanted cardioverters, or an uncontrolled blood clotting disorder were excluded. All participants signed the informed consent document.

**Table 1.** Inclusion and exclusion criteria

**Inclusion criteria:**
Adult males and females legally able and willing to participate in the study and come for follow-up visits
Able and willing to fill out the study forms and to communicate with the investigator
Patient with unilateral or bilateral lumbar facetogenic pain of >6 months’ duration
Patients presenting with 1) a positive response (>70% pain relief) to a previous L1 to L5 lumbar medial branch block and/or 2) a positive response (>70% pain relief) to a previous lumbar facet thermal radiofrequency denervation within the prior 6 months
Average pain score of 4 or higher in the prior month (on a scale of 0 to 10)
**Exclusion criteria:**
Pregnant or breastfeeding patient
Patients younger than 18 or older than 80 years
Patients presenting with neurological deficits (including lumbosacral radiculopathy but not radicular pain)
History of spine surgery
Presence of metal hardware at the lumbosacral spine
Lumbar spine pathology that may increase procedural risk and/or influence symptoms and/or generate unrelated AE (per the discretion of the study principal investigator)
Patients unable to understand and complete the research questionnaires in English or French
Any severe medical condition preventing the patient from safely and effectively being treated in the study or reporting study outcome
Patient with extensive scarring in the skin and tissue overlying the treatment area
Patients enrolled in or planned to be enrolled in another clinical trial during the duration of this research project

### Outcome Assessment

#### Safety

All adverse events (AEs) were captured, including any AE related to the investigational medical device or an untoward medical occurrence during the study period. Participants reported AEs, and then the treating physicians rated the AEs as mild, moderate, or severe. The relation of the AEs to the device and procedure were classified by the treating physicians as very likely or certain, probable, possible, unlikely, unrelated, or unclassifiable.

#### Effectiveness

Data collection included the following questionnaires: the NRS for pain, the Roland-Morris Disability Questionnaire, the Brief Pain Inventory, and the Patient Global Impression of Change. Additional information on analgesic consumption and findings from a targeted neurological examination performed by the investigators were also collected. Follow-up appointments were performed via telephone interviews and office visits, as shown in Table 2. We defined clinical success as either 1) a reduction of two points on the NRS without an increase in the opioid intake or 2) a reduction of opioid intake without an increase in the NRS. Although NRS scores were not limited to low back areas, we considered only pain from the lumbar region as per the Brief Pain Inventory when analyzing the clinical success rates, where possible.

**Table 2.** Study visit and testing schedule, with follow-up appointments performed via telephone interviews and office visits

Study Chronogram	Study Visit and Testing Schedule								
	Screening	Procedure	Follow-Up						End of Study
	V0	Vx	V1 +2 days	V2 +7 days	V3 +14 days	V4 +28 days	V5 +84 days	V6 +168 days	V7 +365 days
	On Site	On Site	*Phone*	On Site	*Phone*	On Site	On Site	On Site	On Site
Informed consent	**X**								
Inclusion and exclusion	**X**	**X**							
Weight and height	**X**								
Physical exam	**X**			**X**		**X**	**X**	**X**	**X**
Review pain treatments	**X**			**X**		**X**	**X**	**X**	**X**
Current pain medications	**X**	**X**	**X**	**X**	**X**	**X**	**X**	**X**	**X**
Questionnaires									
BPI	**X**			**X**		**X**	**X**	**X**	**X**
RMDQ	**X**			**X**		**X**	**X**	**X**	**X**
PGIC				**X**		**X**	**X**	**X**	**X**
NRS			**X**		**X**				
Pregnancy test		**X**							
Patient training		**X**							
Neurolyser XR data		**X**							
Adverse events		**X**	**X**	**X**	**X**	**X**	**X**	**X**	**X**

V = Visit; BPI = Brief Pain Inventory; RMDQ = Roland-Morris Disability Questionnaire; PGIC = Patient Global Impression of Change; NRS = Numeric Rating Scale (for pain).

### Fluoroscopy-Guided HIFU Procedure

**Figure 2.**
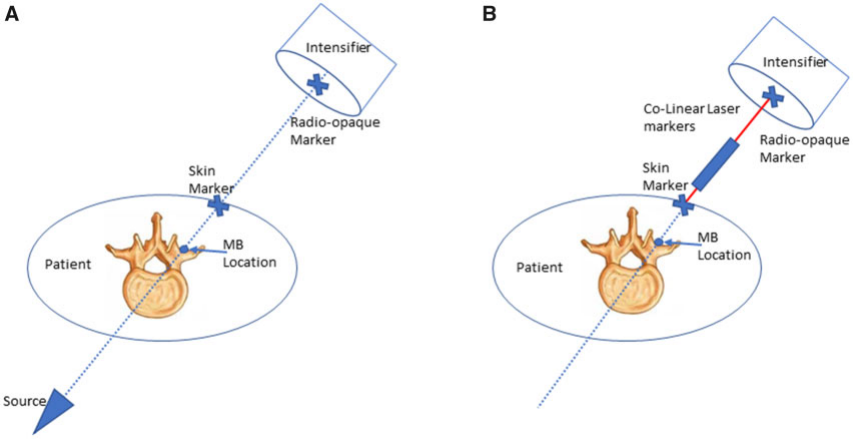
Targeting alignment. **(A)** Placement of the two radio-opaque markers, with the imaginary line starting at the center of the image field of view and ending at the target. **(B)** The colinear laser markers aligned to the center of each of the radio-opaque markers.

**Figure 3.**
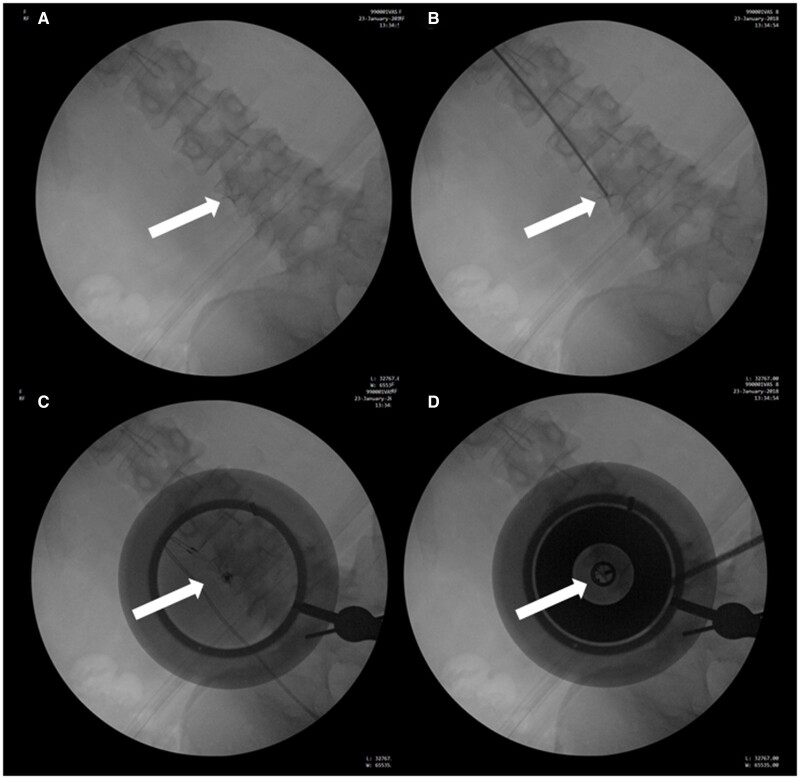
Targeting procedure. **(A)** X-ray image of the lumbar spine with an “X” marker (white arrow), attached to the C-arm image intensifier, positioned over the target area over the MB nerve along the lateral L4 pedicle. **(B)** X-ray image with an opaque rod denoting the skin site over the target area (white arrow), which is then marked with a permanent marker. **(C)** With the use of colinear lasers, the center of translucent mockup cradle is aligned to the mark on the skin, and the center of the “X” marker is aligned on the intensifier (white arrow). For accurate targeting, the laser spot position should be <6 mm from the center of the “X” from the image intensifier marker and <2 mm from the center of the skin marker. **(D)** Central mockup cradle is replaced with the more opaque Neurolyser Transducer, including an x-ray aimed at the center of the transducer (white arrow). The final targeting accuracy confirms the target in the center of the concentric circles.

Participants were instructed to take their regular analgesics up to 1 hour before the sonication on the procedure day. The procedures were performed in a nonsurgical, lead-protected room with mobile C-arm fluoroscopy guidance (GE OEC 9900 (Milwaukee, WI) or Siemens Siremobil Compact L (Munich, Germany)). Participants lay prone on the table with a positioning prop placed under the abdomen to correct lumbar lordosis. The anatomic location of the MB was localized through the use of fluoroscopy and a colinear laser, following the same bony landmarks as conventional RFA (Figure 2). A more detailed description of this targeting procedure is provided below and demonstrated in Figure 3.

1. A radiopaque sticker (2-cm, plus-shaped marker, IZI Medical, Maryland) was placed at the center of the image intensifier, and the C-arm was positioned so the silhouette of the radiopaque sticker overlapped the targeted MB.
2. A mark was drawn on the skin over the target area. The coupling gel pad and transducer cradle were then placed over this mark.
3. A colinear laser was placed inside the cradle and was manually adjusted until the laser pointer aligned at both the radiopaque sticker on the image intensifier and the mark on the skin. This alignment was verified by fluoroscopy and two colinear markers on the tips of the colinear laser pointer.
4. After targeting was verified, the colinear laser was switched with the HIFU transducer, which has two centrally located radiopaque markers (solid metal rings).
5. Final anterior-posterior verification was obtained by finding the anatomic landmark inside the two radiopaque ring-shaped markers.
6. A lateral view of the lumbar spine provided depth verification. The anatomic target had to be found within the acoustic focal spot, with the cradle silhouette used as a reference. After the acoustic focal point location was confirmed to be at the correct depth, the procedure began.

### HIFU Parameters

After the completion of the target planning, the physician performed a verification sonication using 300 J delivered over 20 seconds to ensure that the participant experienced no abnormal sensory or motor symptoms. A tissue-destructive HIFU sonication was then performed with energies of 1,000 J to 1,500 J for 50 seconds per treatment site. With similar technical parameters used, previous data from preclinical, simulation, and cadaver studies showed the expected temperature at the targeted area to reach 100 degrees Celsius [12]. On the basis of histology from pigs euthanized at 1 week after the procedure, the average lesion size was 16 × 8.8 mm in the shape of a “Hershey’s Kiss” chocolate, with the flat base along the bony surface [12]. During and after each sonication, participants were prompted to vocalize any significant discomfort, pain, radiating symptoms, or other sensations. This feedback helped to avoid thermal damage to unrelated anatomic structures and tissues. Bilateral procedures were clinically indicated in some participants. Initially, three levels were ablated unilaterally at L3, L4, and L5 [13], and the contralateral side was attended 2 weeks later. After the first four participants underwent the study procedure with no significant device- or procedure-related AEs, the protocol was amended to allow bilateral procedures during the same session.

### Study Oversight

This study was conceived by the FUSMobile team, including AH, RA, JFA, JP, and SL. Data were independently monitored by McGill University’s clinical research department and reported to Health Canada. All authors collected and analyzed data. All authors had access to the data and vouched for its accuracy. Financial support was provided by FUSMobile and the Focused Ultrasound Foundation, Charlottesville, VA.

### Statistical Analysis

Because of the small sample size and attrition, only descriptive statistical analysis was implemented on the basis of the average NRS per datapoint. Participants who had an alternative therapy within the study period were excluded from further analysis. No additional statistical analyses beyond descriptive statistics were done because of the small number of participants. The secondary outcomes were recorded and presented in a narrative form.

## Results

Ten participants with a mean age of 62.5 years (range 36 to 76) and an average body mass index of 33.6 kg/m2 (range 27.7 to 41.6) were recruited from the clinic pool of patients. Four participants were male and six were female. One participant left the study after 1 month because of lack of response. One participant underwent two RFA procedures because of increased pain shortly before the 6-month follow-up visit. Two additional participants underwent RFA between the 6-month and 1-year follow-up visits because of increased pain. We did not include further NRS scores for the participants that exited the study or had an alternative therapy from that time point forward, because their subsequent pain scores would reflect their clinical status after RFA and thus would be unrelated to the study procedure. Those participants were considered treatment failures.

### Primary Outcome

There were no immediate procedural or device-related AEs. All procedures were well tolerated and completed without interruption, except for one sonication during which the participant reported a temporary intense but nonpainful sensation in the lower back. After the device had been repositioned, subsequent sonications were successful. A review of the fluoroscopy images showed that the device was likely positioned too medially, which might have irritated the joint capsule. No abnormal findings in clinical neurological examinations were reported for any of the participants.

### Secondary Outcome

**Figure 4.**
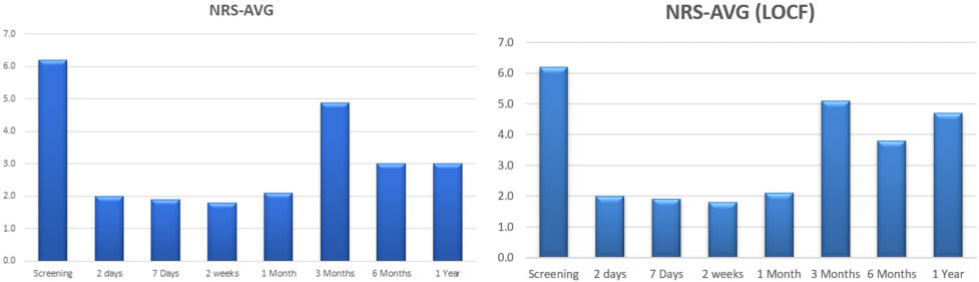
NRS scores over 1 year. **(A)** The average NRS for pain severity decreased from a mean of 6.2 at baseline to 2.1 (n = 10) at 1 month, 4.9 (n = 9) at 3 months, 3.0 (n = 8) at 6 months, and 3.0 (n = 6) at the 12-month follow-up period. **(B)** The average NRS assuming last observation carried forward (LOCF). With this method, pain severity decreased from a mean of 6.2 at baseline to 2.0 at 1 month, 5.0 at 3 months, 3.8 at 6 months, and 4.7 at the 12-month follow-up period.

**Figure 5.**
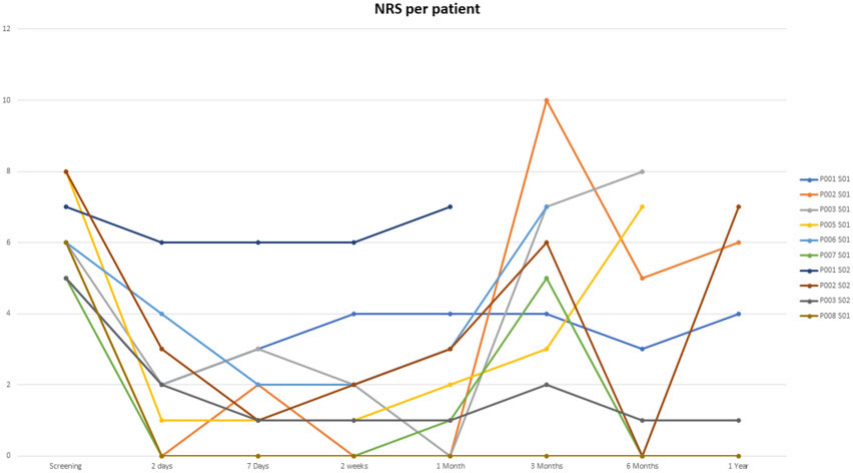
Time plot for each individual NRS score.

**Figure 6.**
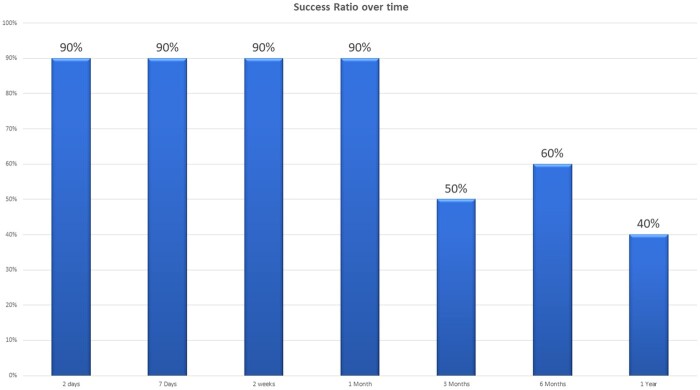
Clinical success ratio over time. Clinical success rates at the 1-, 3-, 6-, and 12-month follow-up periods were 90% (n = 10), 50% (n = 10), 60% (n = 10), and 40% (n = 10), respectively.

**Figure 7.**
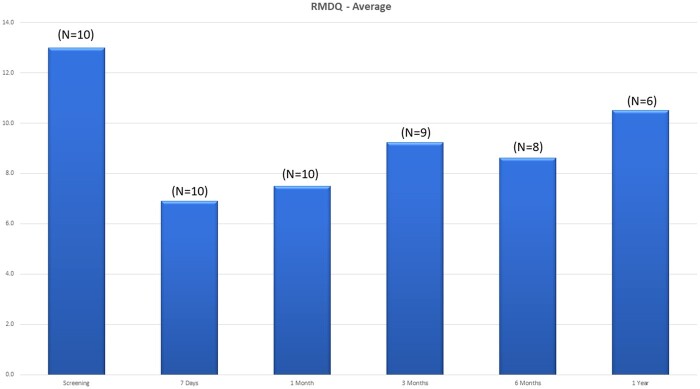
Roland-Morris Disability Questionnaire scores over time.

**Figure 8.**
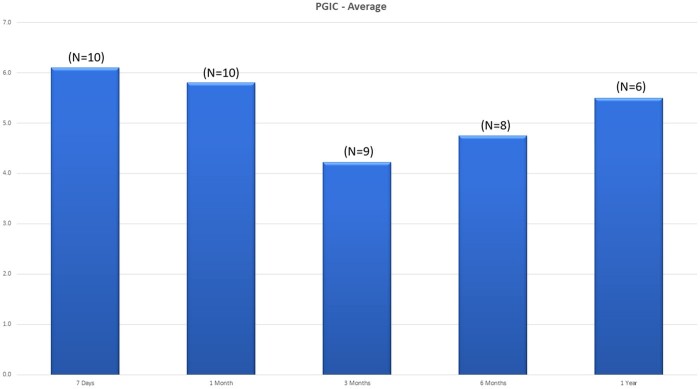
Patient Global Impression of Change scores over time.

The average NRS decreased from a mean of 6.2 at baseline to 2.1 (n = 10) at 1 month, 4.9 (n = 9) at 3 months, 3.0 (n = 8) at 6 months, and 3.0 (n = 6) at the 12-month follow-up period (Figure 4A). For the average NRS with last observation carried forward (LOCF), pain severity decreased from a mean of 6.2 at baseline to 2.0 at 1 month, 5.0 at 3 months, 3.8 at 6 months, and 4.7 at the 12-month follow-up period (Figure 4B). Each individual NRS over time is plotted in Figure 5. Clinical success rates at the 1-, 3-, 6-, and 12-month follow-up periods were 90%, 50%, 60%, and 40%, respectively (Figure 6). Figures 7 and 8 represent the changes over time in the average Roland-Morris Disability Questionnaire and Patient Global Impression of Change scores, respectively.

The average NRS score during sonication was 4.1, which decreased to 1.2 at 30 minutes after the procedure. Three of the 10 participants were taking opioids at baseline. Of these, one discontinued the drug and did not resume taking it during the study, one reduced the dose, and one consumed more.

## Discussion

This clinical pilot study provides preliminary safety and possible effectiveness data for a fluoroscopy-guided portable HIFU device. All 10 participants tolerated the procedure well, with no device- or procedure-related significant AEs at the 1-year follow up. The initial data showed a possible clinical effect at 1 month, which diminished at 3 months, remained stable at 6 months, and then slightly further declined at 12 months. The 6- and 12-month results were comparable to those reported after routine RFA [13]. Other variables, including the Roland-Morris Disability Questionnaire, Brief Pain Inventory, and Patient Global Impression of Change, demonstrated comparable trends in improvement. An increase in pain at 3 months was unexpected. It could be related to small sample size or a diminished expectation-related benefit. Indeed, three participants exited the study between 3 and 6 months, and their NRS scores contributed to the elevated average pain score of the entire group.

Prior technical validation analyses and in vivo preclinical studies in a large animal model in which this fluoroscopy-guided HIFU system was used provided evidence for the safe and effective ablation of the MB in the present study [12, 14].

With further study and optimization, a noninvasive neural ablation procedure offers potential benefits over conventional RFA. Study participants experienced minimal procedural discomfort, required no sedation, and reported no postprocedural aggravation of pain. Pain scores during sonication were mild to moderate and decreased to mild to none within 30 minutes of the procedure. The average NRS scores at 2 and 7 days after the procedure were 2 and 2.2, respectively. In addition to minimal procedural pain and a fast reduction in pain, other potential benefits were identified. For instance, noninvasiveness eliminates the risk of infection and the need for an aseptic technique. Furthermore, current clinical guidelines recommend the discontinuation of anticoagulants before invasive intermediate- and high-risk spinal procedures [15,16]. By ethical review board request, patients on anticoagulants were excluded from the study, although future research and clinical application might not require the discontinuation of anticoagulant and antiaggregant drugs. Also, the acoustic energy used in HIFU does not interact with implanted electrical devices, such as pacemakers, cardioverters, and neurostimulators. Any wearable metallic items or external devices (e.g., hearing aids) are permitted. Thus, HIFU ablation should be a safe treatment option for these individuals. Finally, there is potential to decrease radiation exposure compared with RFA because of the use of optical navigation, but this remains to be demonstrated, and further studies are warranted.

### Study Limitations

This clinical pilot trial has several limitations. It is likely that the inclusion criteria did not adequately identify participants with an isolated zygapophyseal joint disease. At least two participants probably did not have zygapophyseal joint pain, because their subsequent RFAs also failed to improve the symptoms. The inclusion criteria, such as allowing the recruitment of relatively young individuals, those with a baseline NRS of 4, and those with clinical response to only one diagnostic block, were clinically pragmatic yet scientifically lenient. Canadian universal health care insurance does not require diagnostic blocks. Therefore, most practitioners rely on clinical features and single diagnostic blocks, and some perform RFA without any diagnostic blocks. Future studies will include patient selection criteria that are based on the best clinical evidence for RFA and HIFU [17, 18]. Although the present study was conducted with a small sample size and no control group, reporting of the first experience shapes future research endeavors and clinical implementation.

## Conclusion

Using a fluoroscopy-guided HIFU device for MB neurotomy resulted in no significant device- or procedure-related AEs and produced clinical success rates that are comparable to conventional RFA.

## Authors’ Contributions

The planning, conception, and design of this study were performed by JP , MG, SL, AH, RA, and YS. The study was conducted by JP, MG, and YS, who also acquired the data and reported the results. Data analysis and interpretation were performed by AH, RA, SL, JC, and JFA. AH and RA provided technical support.

## Notes

Funding sources: This study received funding from Focused Ultrasound Foundation (Charlottesville, VA) and FUSMobile (Alpharetta, GA ).

Disclosure and conflicts of interest: AH and RA are the founders of FUSMobile. JFA is a member of the scientific advisory board of FUSMobile and holds an option to buy ordinary shares. SL holds shares and ordinary options.

Trial registration: ClinicalTrials.gov: NCT03321344.
